# The Ontogeny of “Twitter” Calls in White‐Faced Capuchins (*Cebus imitator*): Usage, Context, and Acoustic Structure

**DOI:** 10.1002/ajp.70069

**Published:** 2025-08-26

**Authors:** Nicole Guisneuf, Juan Carlos Ordoñez, Marcela E. Benítez, Thore J. Bergman

**Affiliations:** ^1^ Department of Psychology University of Michigan Ann Arbor Michigan USA; ^2^ Capuchins at Taboga Research Project Taboga Forest Reserve Guanacaste Costa Rica; ^3^ Department of Anthropology Emory University Atlanta Georgia USA; ^4^ Department of Ecology & Evolutionary Biology University of Michigan Ann Arbor Michigan USA

**Keywords:** behavior, developmental plasticity, vocal flexibility, Vocalizations

## Abstract

In stark contrast to our own highly plastic communicative abilities, nonhuman primate vocalizations were historically considered fixed and innate, with very little ability to learn or modify vocal signals. However, recent studies indicate that primate vocalizations do show evidence of developmental plasticity, most notably in their context and usage. We build on these studies by investigating developmental changes in one of the most common calls of white‐faced capuchins (*Cebus imitator*), the twitter. Specifically, we examined the behavioral context around twitter vocalizations in a wild population of white‐faced capuchins in the Taboga Forest Reserve, Costa Rica. We analyzed the use of twitters in relation to behavioral state (social, foraging, travel, or resting), immediate context (approaching and leaving others), and specific social behaviors (grooming and aggression). Immatures (infants and juveniles) twitter primarily in a social state, while adults twitter primarily in a foraging state. The twitters produced by immatures were more closely associated with approaching other group members within 1 m, compared to adults. This contextual shift with development from social to foraging prompted us to also look for corresponding acoustic changes. However, the acoustic properties of twitters were not related to context or age, with the one exception that adult twitters were longer than those from immatures. Overall, our results suggest that the twitter is a call with multiple functions, with a shift in usage from social to foraging contexts across development. This adds to the growing evidence of flexibility and learning in primate vocal communication.

## Summary

- **Developmental vocal plasticity**: Evidence of developmental changes in twitter vocalizations of white‐faced capuchins, indicating flexibility and learning.
- **Contextual shift**: Infants and juveniles twitter more frequently in social contexts compared to adults. Shift in twitter usage from social to foraging contexts across development.

## Graphical abstract

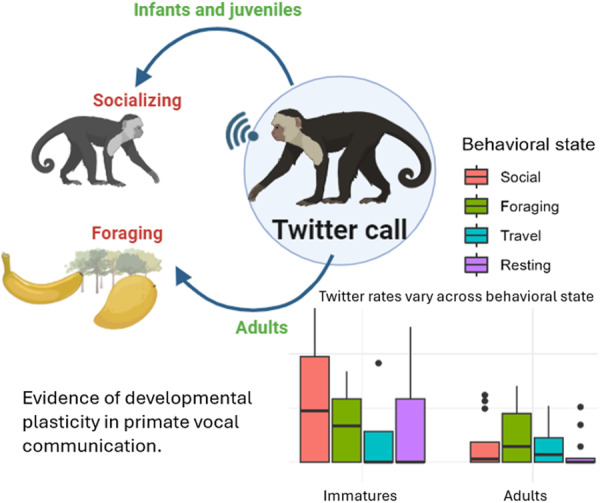

## 1 Introduction

Nonhuman primates are particularly relevant for understanding the origins of language. They are our closest living relatives and have vocalizations that are diverse, and important for maintaining social relationships (reviewed in Fedurek and Slocombe 2011). However, a central puzzle in human origins is that human language relies extensively on learning and plasticity, but we see comparatively little flexibility and vocal control in nonhuman primates (Cheney and Seyfarth 2018; Bergman et al. 2019). Some have proposed that birds are a better model for understanding the importance of learning and plasticity in vocal communication based on convergent similarities in these features (Wilbrecht and Nottebohm 2003; Nowicki and Searcy 2014; Hyland Bruno et al. 2021). Avian studies certainly help us understand the nature of vocal plasticity but are limited in what they can tell us about the evolutionary pathway to language. A precise understanding of the extent of vocal flexibility in nonhuman primates can help us understand both the starting point and the extent of evolution of plasticity and learning that occurred in the human lineage but we currently lack this precision. Indeed, the amount of flexibility and learning that occurs in primate vocal communication has been heavily debated (Zuberbühler et al. 2009; Fedurek and Slocombe 2011).

Primate vocalizations were originally described as largely innate and inflexible, particularly with regard to acoustic structure (Tomasello 2008; Zuberbühler 2017; Lemasson et al. 2013). For example, squirrel monkeys (*Saimiri sp.*) raised in isolation still develop all call types in the species' specific vocal repertoire (Hammerschmidt et al. 2001; Zimbler‐DeLorenzo and Stone 2011). Similarly, even with no experience with predators, captive brown capuchins (*Sapajus apella*) still develop recognizable species‐specific alarm calls (Boinski et al. 1999). This may indicate that there is no learning involved, and primate vocalizations are closely linked to the internal state of the caller (Jarvis 2019). If we accept this apparent inflexibility in vocal development, this suggests our own highly plastic vocal abilities represent a uniquely derived human ability.

Yet, studies have shown remarkable flexibility and ontogenetic plasticity in the usage and context of nonhuman primate calls, with evidence across primate species that this aspect of communication is more flexible than the production of the vocal repertoire itself (Hauser 1989; Liebal et al. 2013; Briseno‐Jaramillo et al. 2018). For example, young vervet monkeys (*Chlorocebus pygerythrus*) and sooty mangabeys (*Cercocebus atys*) will alarm call for a wide range of species (including those that pose no danger), taking multiple years to learn the appropriate context for these different alarm calls (Seyfarth and Cheney^1986^; León et al. 2023). Moreover, social interactions (such as call exchanges with parents) and social reinforcement (such as adults responding to alarm calls given in the correct context) facilitate the development of adult‐like calls and usage (vervet monkeys: Seyfarth and Cheney 1986; Cheney and Seyfarth 2018; marmosets: *Cebuella pygmaea*: Snowdon 2018; *Callithrix jacchus*, Ghazanfar et al. 2019). These studies all indicate that nonhuman primate vocalizations develop their full expressions in a social context and with a learning component.

Studies of the ontogeny of nonhuman primate vocalizations can help us understand the role of plasticity and learning in nonhuman primate vocalizations. In this study, we examine the development of the twitter, a common call that might have a contact function or more general affiliative function, in wild white‐faced capuchin monkeys (*Cebus imitator*). Capuchin monkeys are highly social (Fedigan and Bergstrom 2010; Perry 2012), living in groups averaging around 17 individuals comprised of immigrant adult males, natal adult females, and their offspring (Fedigan and Jack 2011). They have complex social relationships that involve extensive cooperation and coalitionary aggression (Perry et al. 2003) and they exhibit a wide range of vocal signals (Gros‐louis et al. 2008). Capuchins exhibit social learning both in the wild with *Cebus* species (Perry et al. 2003; Perry et al. 2017) and in captivity with *Sapajus* species (Coelho et al. 2015; Steinberg et al. 2022). We seek to identify whether their social learning abilities also extend to vocal behavior.

Contact calls are a particular call type that allow group members to coordinate and facilitate contact with kin and group members (Kondo and Watanabe 2009). They are ideal calls for investigating ontogeny for several reasons. First, considerable data exist on their acoustic structure and context of production (Kondo and Watanabe 2009). Second, contact calls are potentially more developmentally complex and flexible than other call types because they mediate ongoing interactions between group members (Elowson et al. 1992). Other call types, such as isolation or alarm calls, have a high immediate survival benefit and so are likely to be more fixed and show fewer changes during development (Elowson et al. 1992). The most common contact call for capuchins is the twitter (also referred to as a trill). This contact call is one of over 17 distinct call types in the white‐faced capuchin vocal repertoire (Gros‐louis et al. 2008). Twitters appear to show signs of developmental plasticity since they are used in multiple contexts, by multiple age groups (Boinski 1993; Gros‐Louis 2002). Adults often use twitters to maintain contact and cohesion within a group and to initiate troop movement (Boinski 1993; Gros‐Louis 2002; Boinski and Campbell 1995). However, particularly among infants, twitters tend to be used during close‐range social interactions and are reported to increase affiliative interactions, such as receiving grooming, and decrease aggression (Gros‐louis et al. 2008; Gros‐Louis 2002). Juveniles of both sexes produce twitters, but adult males twitter very little compared to females (Gros‐louis et al. 2008). These findings provide indirect evidence that call usage may transition with age. Given this potential flexibility in usage, the twitter is also an ideal call to identify any acoustic shifts across ontogeny. A recent study of bearded capuchins assessed six call types during development and found acoustic changes which, although partially explained by growth and maturation, suggest that learning may play a role, especially in relation to temporal parameters (Ferreira and Izar 2023).

Here, we conduct a cross‐sectional study, to examine the function and form of twitters across development. To do this, we examined the function of twitters, looking at the context in which they are used. We predicted that immatures (infants and juveniles) would use twitters in social interactions, whereas adults would primarily use them to coordinate group movement. We then examined the form of twitter calls across age groups, investigating age differences in acoustic parameters, and exploring the possibility that there are different subtypes of twitters, with consistent acoustic differences, associated with different contexts.

## 2 Methods

### 2.1 Study Site and Species

This study was approved by the Institutional Animal Care and Use Committee and adhered to the American Society of Primatologists Principles for the Ethical Treatment of Nonhuman Primates. Research was conducted at the Taboga Forest Reserve, a tropical dry forest approximately 66 km Southeast of Liberia, in the Guanacaste province of Costa Rica, locatedat 10°20'06”N, 85°08'56”W. The elevation at the study site is approximately 51 meters above sea level. The Reserve has a high density of white‐faced capuchins compared to other sites in Costa Rica, despite high levels of habitat fragmentation (Tinsley Johnson et al. 2020). For this project, we focused on two habituated groups of capuchins with the most consistent data collection and largest sample of infants and juveniles. Our sample consisted of 31 individuals (breakdown of age groups in Table 1), though not all individuals were present in the group for the entire data collection period. In this study, we focused on twitter vocalizations and social behaviors of each age group. Monkeys entered our sampling only after 6 months of age, and they were classified as infants until they started to become independent from the mother at 2 years. For those born during the time of the project, which started in 2017, we have dates of birth within a few days, and for those born before 2017, we have an estimated date of birth based on size and secondary characteristics. Females were considered juveniles from 2 years old until they reached sexual maturity and had their first offspring (~ 6 years), and after this were classified as adults. Males were considered juveniles from 2 years old until 8 years, subadults between 8 and 10, and adults after 10 years (Gros‐Louis 2002). Since we did not have a female category of subadult and only 2 male subadults, we decided to exclude these from our analyses to keep age groups consistent. Because our sample size for infants was small, we combined infants and juveniles together in the analyses with age as a categorical variable.

**Table 1:** Sample composition by age and sex class.

	Infants	Juveniles	Adults	Total
Number of males	3	3	6	12
Number of females	2	9	8	19
Total obs minutes; males	680	1040	3250	4970
Total obs minutes; females	50	3260	5030	8340
Total observation minutes	730	4300	8280	13,310
Male recordings	18	21	4	43
Female recordings	19	89	180	288
Total recordings	37	110	184	331

### 2.2 Behavioral Observations

We included behavioral data from January 2019 to March 2020, and again from January to December 2021 using 10‐min continuous focal observations (Altmann 1974), recorded using the Animal Observer program (Caillaud 2017) on an iPad. During the focal, we recorded all changes in behavioral state (rest, forage, social, or travel), and we used this information to understand the broad context in which twitters occurred. We also continuously recorded all behaviors, including any vocalizations. We used this information to look at specific behaviors (approach, leave, groom, aggression) the minute before and after a twitter was produced to understand what was happening in association with these vocalizations. We chose a 1‐min period because this was narrow enough to be associated with a twitter, but broad enough to capture behaviors that may not be an instantaneous response. To test differences in context due to age, we compared specific behaviors surrounding twitter calls to baseline twitter rates throughout the focal period. If twitters occurred within 1 min of another twitter, we only included the first one to ensure independence of samples.

### 2.3 Acoustic Recordings

We opportunistically recorded twitter vocalizations using a Zoom H5 recorder and either a Sennheiser ME600 shotgun microphone or a Wildtronics parabolic microphone. We collected ad lib recordings, using the pre‐record function on the recorder. Recordings were taken at a maximum distance of 15 m from the caller and the acoustic quality of the call was further examined by creating spectrograms using Avisoft software (Avisoft Bioacoustics 2023). Only the highest quality recordings with minimal background noise were included in the analyses. For each recording, we noted the individual ID of the caller, and the behavioral state, which we classified as either foraging, socializing, traveling or resting. We were particularly interested in looking at twitters during social and travel states since the previous literature suggests that these may relate to different functions of twitters.

### 2.4 Statistical Analyses

#### 2.4.1 Is Age a Predictor of Twitter Rates?

All statistical analyses were performed using “R,” version 4.4.1 (R Core Team 2023). To assess whether age was a predictor of the number of twitter vocalizations, we conducted a negative‐binomial mixed model using the glmmTMB package in R (Brooks et al. 2017), which is the most appropriate model type due to excess zeros in the outcome variable, and overdispersion in the data. We used twitter rates per 10‐min focal as the outcome variable. We included age (standardized), and sex as independent variables, and individual as a random effect to control for individual variation (formula: twitter rate ~ age (standardized) + sex + (1 | ID)). We then compared the AIC of each model (intercept only, age only, age and sex, age‐sex interaction) to determine the best fit and which variables were best predicting the number of twitters.

### 2.5 Does Behavioral State Affect the Number of Twitters Differently Across Age Groups?

Because of overall age differences in twitter rates across age groups, we also compared twitter rates within each age group, to assess the distribution of twitters across behavioral state. We created two models (one for immatures, one for adults) using a beta distribution, with twitter rate relative to time spent in that state as the outcome and behavioral state as the predictor (formula: twitter rate ~ behavioral state + sex + (1 | ID)).

### 2.6 Are Twitters Associated With Different Contexts Across Age Groups?

To examine the behavioral context of twitter calls, we looked at behaviors that occurred within 1 min before and after each twitter occurrence, with a focus on social behaviors, since these are potentially interesting for providing insight into what twitter vocalizations are being used for. We investigated approaches (where the focal moves within 1 m of another individual), and leaves (where the focal moves away from another individual who was within 1 m), as an index of close proximity and indicator of potential social interaction. We also investigated specific social behaviors including grooming (both giving and receiving) and aggression (both giving and receiving). We calculated the proportion of twitters associated with each behavior (e.g. number of twitters within 1 min of an approach/total twitters) and used a Mann‐Whitney U Test (Mann and Whitney 1947) to assess differences between adults and immatures. This nonparametric test was appropriate because the data were not normally distributed and consisted of independent groups.

### 2.7 Does Age Affect the Acoustic Structure of Twitter Calls?

**Figure 1:**
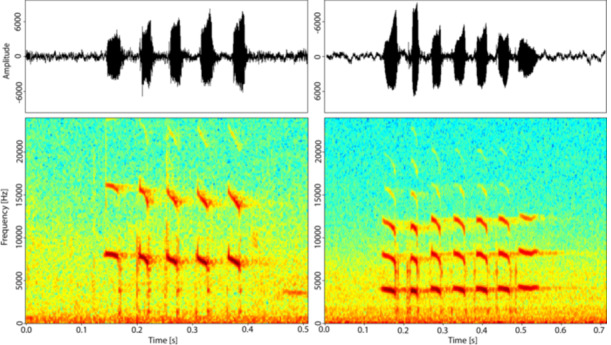
Waveform and spectrogram of two twitter vocalizations. Left: juvenile female. Right: adult female. These were created in R using the “TuneR” package (Ligges et al. 2023), with a sampling rate of 4800 Hz, and a window of 256, nfft of 1024, and overlap of 128.

We created spectrograms of each individual twitter (Figure 1) and, using Avisoft, measured three acoustic parameters. We measured the duration of each call, in seconds, the maximum frequency across the call, in hertz (Hz), and the bandwidth, in Hz (the range of frequencies, difference between the highest and lowest frequencies). Acoustic structure may change with growth, due to an increase in the size of the vocal apparatus. This likely has an effect on frequency‐based parameters, with a larger vocal tract creating a lower frequency call (Taylor and Reby 2010). However, call duration is less closely linked to body size, and therefore any differences in duration may suggest a difference beyond growth and point to potential developmental changes. We created linear mixed effect models, using the lme4 package, to assess whether age is a predictor of each of the three acoustic parameters. We included individual as a random factor to control for individual differences. We built a set of models for each outcome variable: duration, maximum frequency and bandwidth, hierarchically adding age, sex, context, and group as the predictor variables (formula: duration ~ age + sex + context + group + (1 | ID)).

## 3 Results

### 3.1 Age Is a Significant Predictor of Twitter Vocalizations

**Figure 2:**
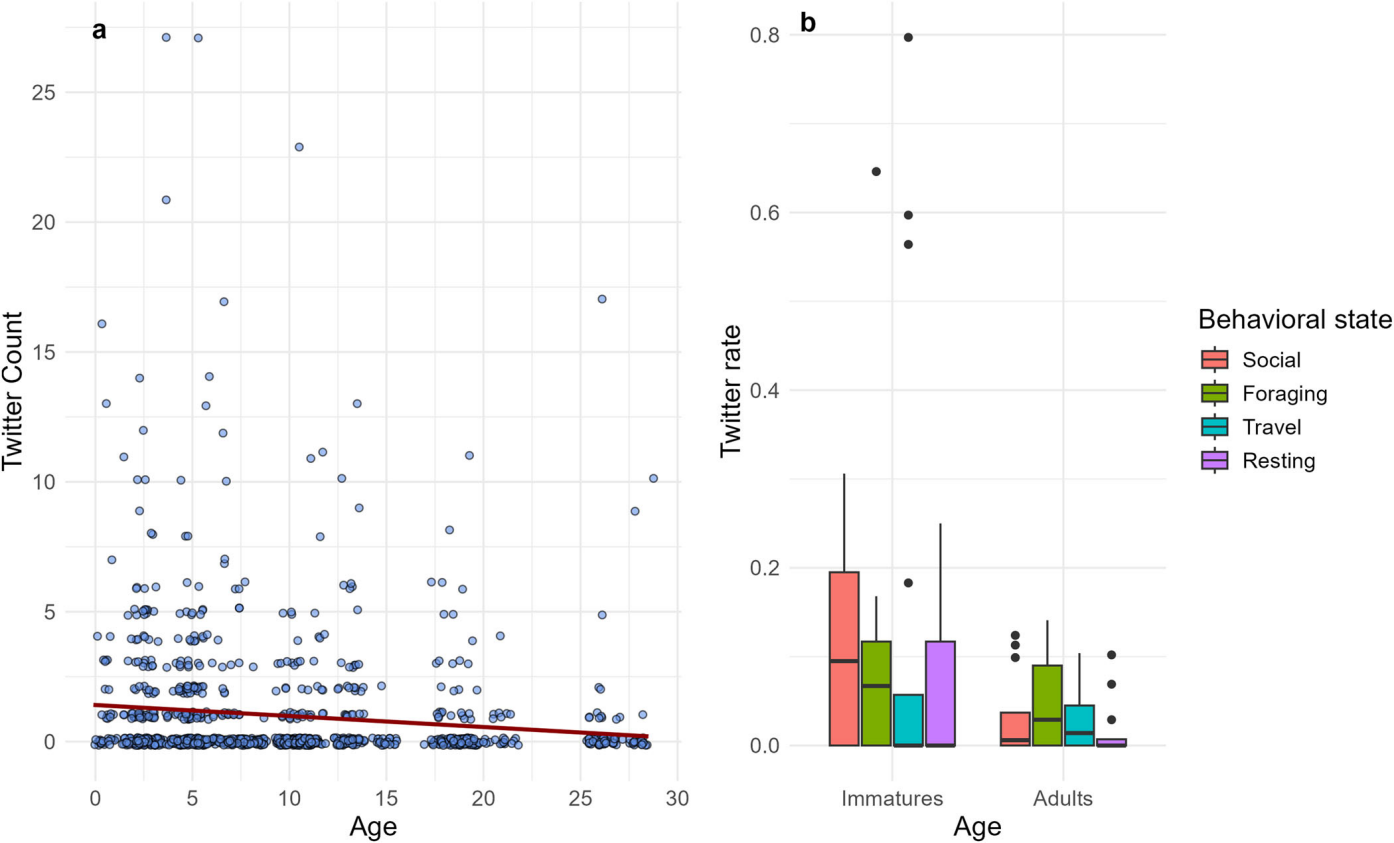
(a) Scatterplot showing the relationship between twitter count for each 10‐min focal, and age in years. (b) Distribution of twitter rate in each behavioral state. Rates were calculated using twitter count in each state/the total time spent in each state.

In total, we included 221 h of observation time and 946 twitter occurrences (mean per individual: 42.21, range: 0–139). We found significant associations between twitter count and age (estimate = −0.399, SE = 0.093, z = −4.281, *p* < 0.0001, Confidence intervals: −0.58, −0.22: Table 2). For every year increase in age, the number of twitter vocalizations recorded in a 10‐min period decreases by approximately 0.06. In other words, the number of twitters in a focal observation is expected to decrease as age increases (Figure 2a). Sex was also an important predictor of twitter production (Estimate = −0.650, SE = 0.188, *z* = −3.453, *p* < 0.001, Confidence intervals: −1.02, −0.28) such that females twittered 0.62 times more than males. This model fit better than intercept only, age only, and an age‐sex interaction, as it had the lowest AIC value and majority of the weight.

**Table 2:** Results of multivariate negative binomial regression analysis predicting twitter count from each 10‐min focal observation.

Effect	Estimate	SE	95% CI		Z value	*p* value
Twitter rate						
Intercept	0.02	0.135	−0.24	0.29	0.149	0.88
Age (std)	−0.399	0.093	−0.58	−0.22	−4.281	< 0.001***
Sex (Male)	−0.65	0.188	−1.02	−0.28	−3.453	< 0.001***

*** *p* < 0.001.

### 3.2 Behavioral State Affects Number of Twitters Differently Across Age Groups

Among immatures, we found no significant association between twitter rate and behavioral state (Table 3). However, social state was trending in a positive direction (*p* = 0.09), suggesting that immatures are more likely to twitter in a social state, relative to resting state. Neither sex nor an interaction between behavioral state and sex improved model fit. Among adults, we found that twitter rates were significantly higher in a foraging state (estimate = 1.08, SE = 0.41, z = 2.65, *p* = 0.008, confidence intervals: 0.28, 1.89) and a travel state (estimate = 0.83, SE = 0.41, *z* = −2.24, *p* = 0.04, confidence intervals: 0.04, 1.63), compared to a resting state (Table 3). The odds of twittering in a foraging state are 2.6 times higher compared to the resting state. The odds of twittering in a travel state are 2.29 times higher than in a resting state. We also found that including sex in the model improved model fit (lowest AIC and highest weight), and that male twitter rates are significantly lower compared to females. The odds of twitters for males are 0.69 times lower than for females. Altogether, our results suggest that immatures not only twitter at higher rates than adults, but twitter primarily in social states, while adults twitter mostly during foraging and travel (Figure 2b, Supporting Information S1: Table S1). The time spent in each behavioral state also varied with age, and sex (Table 4). Most notably, adult males spent less time socializing than adult females and immatures of both sexes. However, even though adult females and immatures spend a comparable time socializing, immatures still twitter more.

**Table 3:** Results of the effects of behavioral state on twitter rates within each age group.

Effect	Estimate	SE	95% CI		*Z* value	*p*
Immatures						
Intercept	−2.703	0.378	−3.444	−1.963	−7.157	< 0.001***
Foraging state	0.452	0.375	−0.284	1.187	1.204	0.229
Social state	0.641	0.378	−0.099	1.381	1.697	0.09
Travel state	0.032	0.371	−0.696	0.76	0.086	0.931
Adults						
Intercept	−4.111	0.455	−5.002	−3.22	−9.039	< 0.001***
Foraging state	1.084	0.41	0.282	1.887	2.648	0.008**
Social state	0.652	0.414	−0.159	1.463	1.577	0.115
Travel state	0.83	0.406	0.035	1.625	2.045	0.041*
Sex ‐ male	−1.156	0.517	−2.169	−0.142	−2.235	0.025*

* *p* < 0.05

** *p* < 0.01

*** *p* < 0.001.

**Table 4:** Mean time (proportion of a 10 min focal observation) spent in each behavioral state, the total twitter count and mean number of twitters per focal.

State	Age	Sex	Average time spent	Total twitters	Average twitters
Social	I&J	*M*	0.373 ± −0.144	75	10.7 ± 12.5
	I&J	*F*	0.341 ± 0.227	170	17 ± 27.5
	A	*M*	0.19 ± 0.085	2	0.33 ± 0.52
	A	*F*	0.369 ± 0.196	77	9.62 ± 12.5
Foraging	I&J	*M*	0.454 ± 0.097	61	8.71 ± 8.73
	I&J	*F*	0.455 ± 0.23	192	19.2 ± 25.9
	A	*M*	0.373 ± 0.077	33	5.5 ± 12.5
	A	*F*	0.435 ± 0.173	205	25.6 ± 22.5
Travel	I&J	*M*	0.071 ± 0.029	4	0.57 ± 0.98
	I&J	*F*	0.043 ± 0.034	46	4.6 ± 7.6
	A	*M*	0.16 ± 0.123	2	0.33 ± 0.52
	A	*F*	0.076 ± 0.028	18	2.25 ± 2.76
Resting	I&J	*M*	0.101 ± 0.15	6	0.86 ± 1.21
	I&J	*F*	0.164 ± 0.18	37	3.7 ± 5.91
	A	*M*	0.275 ± 0.11	3	0.5 ± 0.84
	A	*F*	0.119 + 0.08	37	4.62 ± 7.5

### 3.3 Twitters Are Associated With Close Social Interactions for Immatures Only

**Figure 3:**
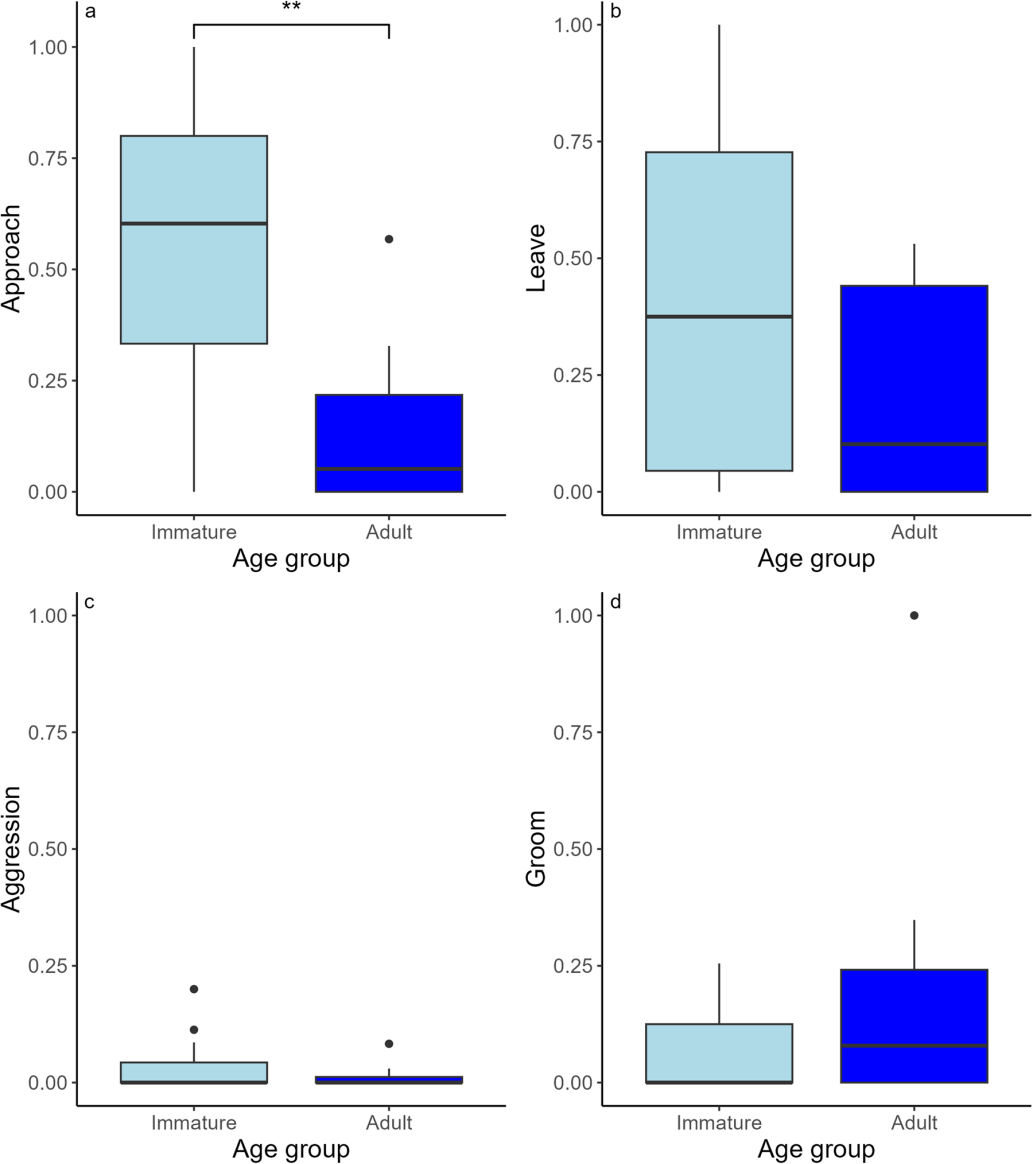
Proportion of twitters that happened within 1 min of given behavior. (a) Approach: moving to within 1 m of another group members; (b) leave: moving out of 1 m of another group member; (c) giving or receiving aggression (contact and noncontact); (d) giving and receiving grooming. Stars indicate significance of the Mann‐Whitney U Tests comparing immatures and adults. For approach, *W* = 101, *p* = 0.007**. Proportions were calculated by: number of twitters with behavior 1 min before or after/total number of twitters by each individual. This was then used to compare the two age groups.

*Approaching and leaving others*: Twitters uttered by immatures were disproportionately associated with an approach to within 1 meter of another individual, compared to twitters uttered by adults: Mann‐Whitney U: W = 28.5, *p* = 0.004. This means that the proportion of twitters that were associated with approaches, was significantly higher for immatures than for adults (Figure 3a). We found no age differences in the proportion of twitters associated with leaving from within 1 meter of others: Mann‐Whitney U: W = 56, *p* = 0.15 (Figure 3b).

*Social behaviors*: We did not find any age differences in the proportion of twitters associated with aggression: W = 70, *p* = 0.40 (Figure 3c), nor grooming: W = 99, *p* = 0.47 (Figure 3d). For all of these, separating out actor and recipient yielded broadly similar results which we have included in the supplemental material (Table S2).

### 3.4 The Acoustic Structure of the Twitter Shows Some Age‐Related Differences

**Figure 4:**
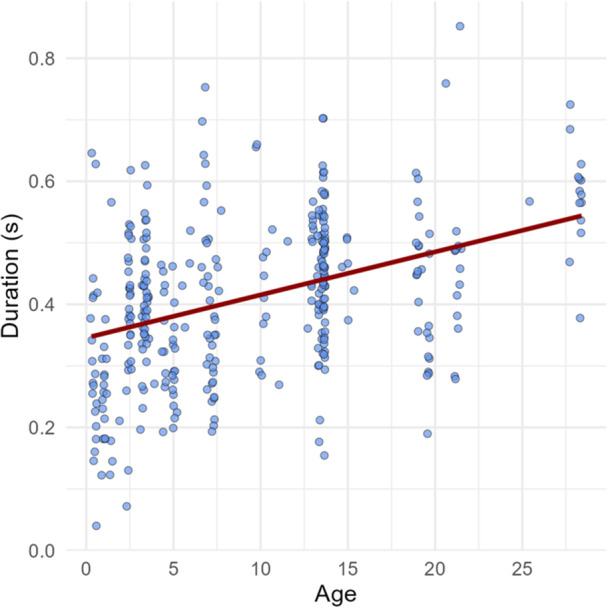
Scatterplot showing the relationship between twitter duration (seconds) and age (years).

We analyzed 331 twitter vocalizations from 27 individuals (immature males, 11 immature females, 2 adult males, 8 adult females, mean number of recordings: 13.52, range: 2–97). We found a significant positive association between age and duration of a twitter vocalization (Estimate = 0.05, SE = 0.02, *z* = 3.578, *p* < 0.01, confidence intervals: 0.024−0.083: Figure 4, Table 5), with mean duration of 0.29 s for infants, 0.39 s for juveniles, and 0.45 s for adults. This means that for each 1‐year increase in age, the duration increases by about 0.007 s. Adding sex, context and group, as well as an interaction between age and context, did not improve model fit.

**Table 5:** Results of the effects of age, sex, and context on acoustic parameters: duration, maximum frequency and bandwidth.

Effect	Estimate	SE	95% CI		*t* value	*p*
Duration						
Intercept	0.406	0.014	0.378		24.85	< 0.001***
Age	0.048	0.013	0.022	0.074	3.58	0.003**
Maximum frequency						
Intercept	11592.92	510.26	10656.784	12495.5	22.72	< 0.001***
Age	−114.06	294.04	−695.404	415.552	−0.39	0.71
Sex	47.27	777.35	−1331.452	1473.994	0.061	0.95
Context resting	−1662.76	1170.5	−4036.863	498.261	−1.42	0.16
Context socializing	−690.24	462.33	−1617.959	164.597	−1.49	0.14
Context traveling	−595.95	449.73	−1448.655	274.409	−1.33	0.17
Group	312.59	615.73	−788.454	1290.859	0.51	0.62
Bandwidth						
Intercept	9562.718	472.2	8733.11	10362.88	20.25	< 0.001***
Age	−260.613	258.55	−778.725	132.839	−1.01	0.368
Sex	−213.326	732.224	−1357.422	1228.146	−0.29	0.776
Context resting	−511.723	1239.26	−2999.031	1764.637	−0.41	0.68
Context socializing	−505.517	486.554	−1563.158	279.9382	−1.04	0.301
Context traveling	−60.559	472.895	−1015.612	746.824	−0.13	0.898
Group	−549.184	542.172	−1369.933	466.3906	−1.01	0.373

** *p* < 0.01

*** *p* < 0.001.

Maximum frequency did not differ significantly with age, and adjusting for other fixed effects had no influence on model fit (Estimate = 16.38, SE = 263.39, *z* = 0.062, *p* = 0.951, confidence intervals: 0.029–0.079). Similarly, bandwidth did not differ significantly with age, and adjusting for other fixed effects had no influence on model fit (Estimate = −260.613, SE = 258.551, *z* = −1.008, *p* = 0.368, confidence intervals: −735.945 to 84.036).

## 4 Discussion

We found that capuchin twitter calls change with age, both in rates and context, which supports the idea that primate communication can be flexible and change across development. Thus, our work directly demonstrates a shift in twitter usage that previous studies of capuchins had suggested based on separate studies investigating twitter usage for travel coordination (Boinski and Campbell 1995), and close‐range interactions (Gros‐Louis 2002). A theory pioneered by Seyfarth and Cheney (1986) is that young primates make mistakes, and slowly learn the correct context for each type of vocalization over time. For example, young vervet monkeys alarm call in a range of contexts, taking up to 3 years to narrow down their alarm calls specifically to predators (Seyfarth and Cheney^1986^). We would therefore expect the highest twitter rates in the youngest monkeys while they make mistakes, with a subsequent decline as they narrow in on the precise context and learn the correct use of the call. Our results are in accordance with this, as twitter rates were highest among infants and decreased with age. However, when we looked for specific contextual differences, our results suggest that this explanation is not the full story. Immatures are not simply using twitters in broader contexts, but in different contexts compared to adults. For immatures, twitters are used mostly in social contexts, while in adults, they are primarily used in foraging and traveling contexts. This would suggest an alternative explanation for the flexibility of twitter calls: that one call serves multiple functions. We found that a higher proportion of immature twitters were associated with moving in close proximity (1 m) to another individual, compared to adults. If infants were simply using twitters incorrectly, this pattern would likely not be seen. For adults, there was no association with approaching others, leaving others, grooming, or aggression. Adults use twitters in foraging contexts and during travel, rather than during close social interactions.

Twitters appear to serve a social function for immature capuchins, perhaps reflecting the different social environment of immatures and adults. It is possible that juveniles and infants use twitters as an appeasement call in ambiguous or unfamiliar interactions or to test the waters with new social partners, whereas adults already have established relationships with other group members. It may also provide a signal of intention for an affiliative interaction, rather than seeking care, as suggested by Gros‐Louis (2002), who found that infants who twitter on approach received lower levels of aggression, and increased subsequent affiliative interactions (Gros‐Louis 2002). In our study, immatures used more twitters during approaches. The ‘twargle’, another call in the white‐faced capuchin vocal repertoire, has been documented as serving a bond testing function for infants, with the majority of twargles being directed towards the alpha or other adult males, potentially capable of committing infanticide (Duchesneau et al. 2022). Because immature monkeys need to test bonds with other group members, whereas adults have usually established their position in the hierarchy, it is possible that twitters also serve a purpose of testing bonds, and we thus see them occur in close‐range social interactions, particularly upon approaching other group members.

For adults, we found an association between twitters and foraging and travel contexts. It may be that these contexts represent situations with social uncertainty so that both immatures and adults use twitters in relation to ambiguous social contexts. However, for adults uncertainty might be more about where other individuals are while for immatures uncertainty is more about the outcome of close interactions. While the nature of the differences between adult and immature twitter use needs further exploration, our results largely followed our predictions for adults. However, we did not expect foraging to be the best predictor of twitters. This is because previous literature suggested only that twitters serve a travel coordination function (Boinski and Campbell 1995), and in most primates, adults typically coordinate group travel (Bezerra et al. 2009). Contact calls related to troop movement are present in other primate species, including spider monkeys (*Ateles geoffroyi*; Briseno‐Jaramillo et al. 2018), Japanese macaques (*Macaca fuscata*; Sugiura 2001) and chimpanzees (*Pan troglodytes*; Mitani and Nishida 1993). Previous capuchin studies found a close association between the use of twitters and the initiation of troop movement in capuchins (Boinski 1993; Boinski and Campbell 1995). In these previous studies, twitters from older group members were nearly exclusively associated with travel (Boinski 1993; Boinski and Campbell 1995). While our results do not entirely follow these findings, since we found a strong association with foraging as well as travel, it should be noted that Boinski and Campbell's (1995) study used a 10‐min period to identify whether the group traveled after the twitter, and we looked at 1 min. For most of our twitters we did not have a full 10 min of observation following a twitter. Furthermore, extending the time window complicates the data and makes it more difficult to know which behaviors are a result of a twitter. It is possible that twitters occurring during foraging indicate an intention to move to a travel state. We therefore investigated the change in behavioral state after a twitter occurs in an exploratory analysis but did not find any evidence that twitters occur disproportionately before a shift into the travel state (data not shown). This opens the possibility that the twitter is simply a broadly used intragroup contact call, used to keep track of group members, and is therefore relevant for foraging contexts as well as travel. Adult males twitter at much lower rates than females, which is consistent with previous literature (Gros‐louis et al. 2008), and further supports a shift in function since immature males twitter at higher rates than adults. Because adult females are more active in the coordination of troop movement (Boinski 1993), it may be more important for females to use this call, compared to males.

We found some age differences in the acoustic structure of twitter calls, with duration increasing with age. This was in line with our predictions and has been observed across a range of primates including common marmosets (Pistorio et al. 2006), chacma baboons (Ey et al. 2007a), and coppery titi monkeys (Clink et al. 2019). This could relate to growth and maturation, including lung capacity, but body size does not necessarily constrain duration in the same way as some frequency‐based parameters because the organs that produce them are soft and not limited by skeletal structures (Taylor and Reby 2010). Neither maximum frequency nor bandwidth were associated with age in this study. This is somewhat surprising given that growth and body size is likely to influence the sound of vocalizations and a decrease in frequency with age has been documented in multiple primate species (Zimmermann and Lerch 1993; Ey et al. 2007a; Ey et al. 2007b), including brown capuchin trill calls (Ferreira and Izar 2023). This is due to a larger vocal tract creating lower frequency vocalizations (Taylor and Reby 2010). Physiological arousal can also impact acoustic structure, for example through changes in respiration rate or tension in the muscles of the vocal folds (Taylor and Reby 2010), and this may therefore be masking any age‐related frequency changes recorded in this study. We also note that, due to the limitations of sample size (27 individuals with recordings), it is possible that some of the age‐related differences could represent individual variation. While all models included individual ID as a random effect to account for repeated measures and baseline differences among individuals, individual‐level variance remained non‐negligible across models. This suggests that although our models controlled for individual differences, our ability to 411 fully disentangle developmental patterns from individual idiosyncrasies is limited by sample size.

Acoustically similar calls with multiple functions exist in other animals (Javan sparrow (*Lonchura oryzivora*) trill calls: Furutani et al. 2018; black‐and‐gold howler monkeys (*Alouatta caraya*), ‘moo’ calls: da Cunha and Byrne 2013). These studies suggest that certain vocalizations may elicit different responses based on contextual cues, rather than inherent acoustic differences. Since twitters are used in a range of contexts, perhaps contextual cues, such as visual information, are more important for producing different responses than the acoustic structure itself. Where previous literature has suggested a lack of development in call production and acoustic structure indicates a lack of learning or flexibility, we argue that using a call in multiple contexts and responding based on subtle contextual cues (similar to graded baboon barks: Fischer et al. 2001) actually indicates high levels of flexibility.

To conclude, our study provides evidence that the usage of twitter vocalizations shifts through development. Specifically for immatures, twitters serve an important social function during close‐range social interactions with other group members. For adults, twitters appear to serve an important function during foraging and travel. Therefore, even in mammals that show little flexibility in call production, there does appear to be some plasticity in the usage of calls (Seyfarth & Cheney 1986). Such flexibility and modification suggest parallels with human communication, which was thought to be somewhat unique. Our results contribute to growing evidence of flexibility & learning in primate communication. Overall, twitters may serve as a contact call, which is broadly affiliative and can include both the closer range social use and a group coordination purpose. The age differences found in this study suggest more complexity within this call type which merits further investigation. Looking at more acoustic parameters and other aspects of usage, such as which individuals the calls are directed at, will help in gaining further insight into call function and where flexibility lies.

## Author Contributions

**Nicole Guisneuf:** conceptualization (lead), data curation (lead), formal analysis (lead), funding acquisition (lead), investigation (lead), methodology (lead), project administration (equal), resources (equal), software (lead), validation (lead), visualization (lead), writing – original draft (lead), writing – review and editing (equal). **Juan Carlos Ordoñez:** data curation (supporting), investigation (supporting), writing – review and editing (equal). **Marcela E. Benítez:** funding acquisition (supporting), project administration (equal), resources (equal), writing – review and editing (equal). **Thore J. Bergman:** conceptualization (supporting), funding acquisition (supporting), project administration (equal), resources (equal), software (supporting), supervision (lead), writing – review and editing (equal), writing – review and editing (equal).

## Supporting information

Supplementary_Information

## Data Availability

The data that support the findings of this study are available from the corresponding author upon reasonable request.

## References

[ajp70069-bib-0001] Altmann, J. 1974. “Observational Study of Behavior: Sampling Methods.” Behaviour 49, no. 3: 227–266. 4597405 10.1163/156853974x00534

[ajp70069-bib-0002] Avisoft Bioacoustics . (2023) Avisoft SASLab Pro (Version 5.3.2) [Computer software]. https://www.avisoft.com/.

[ajp70069-bib-0003] Bergman, T. J. , J. C. Beehner , M. C. Painter , and M. L. Gustison . 2019. “The Speech‐Like Properties of Nonhuman Primate Vocalizations.” Animal Behaviour 151: 229–237. 10.1016/j.anbehav.2019.02.015.

[ajp70069-bib-0004] Bezerra, B. M. , A. da Silva Souto , M. A. B. de Oliveira , and L. G. Halsey . 2009. “Vocalisations of Wild Common Marmosets Are Influenced by Diurnal and Ontogenetic Factors.” Primates 50, no. 3: 231–237. 10.1007/s10329-009-0132-7. 19224328

[ajp70069-bib-0005] Boinski, S. 1993. “Vocal Coordination of Troop Movement Among White‐Faced Capuchin Monkeys, *Cebus capucinus* .” American Journal of Primatology 30: 85–100. 10.1002/ajp.1350300202. 31937019

[ajp70069-bib-0006] Boinski, S. , and A. F. Campbell . 1995. “Use of Trill Vocalizations to Coordinate Troop Movement Among White‐Faced Capuchins: A Second Field Test.” Behaviour 132, no. 11: 875–901. 10.1002/ajp.1350300202.

[ajp70069-bib-0007] Boinski, S. , T. S. Gross , and J. K. Davis . 1999. “Terrestrial Predator Alarm Vocalizations Are a Valid Monitor of Stress in Captive Brown Capuchins (*Cebus apella*).” Zoo Biology 18, no. 4: 295–312. 10.1002/(SICI)1098-2361(1999)18:4<295::AID-ZOO4>3.0.CO;2-5.

[ajp70069-bib-0008] Briseno‐Jaramillo, M. , G. Ramos‐Fernández , T. Palacios‐Romo , J. Sosa‐Lopez , and A. Lemasson . 2018. “Age and Social Affinity Effects on Contact Call Interactions in Free‐Ranging Spider Monkeys.” Behavioral Ecology and Sociobiology 72, no. 192: 1–17. 10.1007/s00265-018-2615-2.

[ajp70069-bib-0009] Brooks, E. , K. Kristensen , J. Benthem , et al. 2017. “glmmTMB Balances Speed and Flexibility Among Packages for Zero‐Inflated Generalized Linear Mixed Modeling.” R Journal 9, no. 2: 378–400. https://journal.r-project.org/archive/2017/RJ-2017-066/index.html.

[ajp70069-bib-0010] Caillaud, D. (2017) Animal Observer (Version 1.2.2) [Mobile App]. App Store. https://apps.apple.com/us/developer/damien-caillaud/id982771482.

[ajp70069-bib-0011] Cheney, D. L. , and R. M. Seyfarth . 2018. “Flexible Usage and Social Function in Primate Vocalizations.” Proceedings of the National Academy of Sciences 115, no. 9: 1974–1979. 10.1073/pnas.1717572115. PMC5834704 29432157

[ajp70069-bib-0012] Clink, D. J. , A. R. Lau , and K. L. Bales . 2019. “Age‐Related Changes and Vocal Convergence in Titi Monkey Duet Pulses.” Behaviour 156, no. 15: 1471–1494. 10.1163/1568539X-00003575.

[ajp70069-bib-0013] Coelho, C. G. , T. Falótico , P. Izar , et al. 2015. “Social Learning Strategies for Nut‐Cracking by Tufted Capuchin Monkeys (*Sapajus spp*.).” Animal Cognition 18, no. 4: 911–919. 10.1007/s10071-015-0861-5. 25800169

[ajp70069-bib-0014] da Cunha, R. G. T. , and R. W. Byrne . 2013. “Age‐Related Differences in the Use of the “Moo” Call in Black Howlers (*Alouatta caraya*).” International Journal of Primatology 34, no. 6: 1105–1121. 10.1007/s10764-013-9718-4.

[ajp70069-bib-0015] Duchesneau, A. , D. G. Edelberg , and S. E. Perry . 2022. “Are Demographic Correlates of White‐Faced Capuchin Monkey (*Cebus capucinus*) ‘Gargle and Twargle’ Vocalization Rates Consistent With the Infanticide Risk Assessment Hypothesis?” American Journal of Primatology 84, no. 1: 1–13. 10.1002/ajp.23344. 34762319

[ajp70069-bib-0016] Elowson, A. M. , C. T. Snowdon , and C. J. Sweet . 1992. “Ontogeny of Trill and J‐Call Vocalizations in the Pygmy Marmoset, *Cebuella pygmaea* .” Animal Behaviour 43, no. 5: 703–715. 10.1016/S0003-3472(05)80195-2.

[ajp70069-bib-0017] Ey, E. , K. Hammerschmidt , R. M. Seyfarth , and J. Fischer . 2007b. “Age‐ and Sex‐Related Variations in Clear Calls of *Papio ursinus* .” International Journal of Primatology 28, no. 4: 947–960. 10.1007/s10764-007-9139-3.

[ajp70069-bib-0018] Ey, E. , D. Pfefferle , and J. Fischer . 2007a. “Do Age‐ and Sex‐Related Variations Reliably Reflect Body Size in Non‐Human Primate Vocalizations? A Review.” Primates 48, no. 4: 253–267. 10.1007/s10329-006-0033-y. 17226064

[ajp70069-bib-0019] Fedigan, L. , and M. Bergstrom . 2010. “Dominance Among Female White‐Faced Capuchin Monkeys (*Cebus capucinus*): Hierarchical Linearity, Nepotism, Strength and Stability.” Behaviour 147: 899–931. 10.1163/000579510X497283.

[ajp70069-bib-0020] Fedigan, L. M. , and K. M. Jack . 2011. “Two Girls for Every Boy: The Effects of Group Size and Composition on the Reproductive Success of Male and Female White‐Faced Capuchins.” American Journal of Physical Anthropology 144, no. 2: 317–326. 10.1002/ajpa.21414. 20979204

[ajp70069-bib-0021] Fedurek, P. , and K. E. Slocombe . 2011. “Primate Vocal Communication: A Useful Tool for Understanding Human Speech and Language Evolution?” Human Biology 83, no. 2: 153–173. 10.3378/027.083.0202. 21615284

[ajp70069-bib-0022] Ferreira, L. G. , and P. Izar . 2023. “Development of Vocal Production in Bearded Capuchin Monkeys: Is There Space for Vocal Learning?” Research Square: 1–25. Preprint. 10.21203/rs.3.rs-2521771/v1.

[ajp70069-bib-0056] Fischer, J. , K. Hammerschmidt , D. L. Cheney , and R. M. Seyfarth . 2001. “Acoustic Features of Female Chacma Baboon Barks.” Ethology 107, no. 1: 33–54.

[ajp70069-bib-0023] Furutani, A. , C. Mori , and K. Okanoya . 2018. “Trill‐Calls in Java Sparrows: Repetition Rate Determines the Category of Acoustically Similar Calls in Different Behavioral Contexts.” Behavioural Processes 157, no. February: 68–72. 10.1016/j.beproc.2018.08.010. 30157464

[ajp70069-bib-0024] Ghazanfar, A. A. , D. A. Liao , and D. Y. Takahashi . 2019. “Volition and Learning in Primate Vocal Behaviour.” Animal Behaviour 151: 239–247. 10.1016/j.anbehav.2019.01.021.

[ajp70069-bib-0025] Gros‐louis, J. J. , S. E. Perry , C. Fichtel , et al. 2008. “Vocal Repertoire of *Cebus capucinus*: Acoustic Structure, Context, and Usage.” International Journal of Primatology 29: 641–670. 10.1007/s10764-008-9263-8.

[ajp70069-bib-0026] Gros‐Louis, J. 2002. “Contexts and Behavioral Correlates of Trill Vocalizations in Wild White‐Faced Capuchin Monkeys (*Cebus capucinus*).” American Journal of Primatology 57: 189–202. 10.1002/ajp.10042. 12210671

[ajp70069-bib-0027] Hammerschmidt, K. , T. Freudenstein , and U. Jürgens . 2001. “Vocal Development in Squirrel Monkeys.” Behaviour 138, no. 9: 1179–1204. 10.1163/156853901753287190.

[ajp70069-bib-0028] Hauser, M. D. 1989. “Ontogenetic Changes in the Comprehension and Production of Vervet Monkey (*Cercopithecus aethiops*) Vocalizations.” Journal of Comparative Psychology 103, no. 2: 149–158. 10.1037/0735-7036.103.2.149.

[ajp70069-bib-0029] Hyland Bruno, J. , E. D. Jarvis , M. Liberman , and O. Tchernichovski . 2021. “Birdsong Learning and Culture: Analogies With Human Spoken Language.” Annual Review of Linguistics 7: 449–472. 10.1146/annurev-linguistics-090420-121034.

[ajp70069-bib-0030] Jarvis, E. D. 2019. “Evolution of Vocal Learning and Spoken Language.” Science 366, no. 6461: 50–54. 10.1126/science.aax0287. 31604300

[ajp70069-bib-0031] Kondo, N. , and S. Watanabe . 2009. “Contact Calls: Information and Social Function.” Japanese Psychological Research 51, no. 3: 197–208. 10.1111/j.1468-5884.2009.00399.x.

[ajp70069-bib-0032] Lemasson, A. , M. Guilloux , Rizaldi , S. Barbu , A. Lacroix , and H. Koda . 2013. “Age‐ and Sex‐Dependent Contact Call Usage in Japanese Macaques.” Primates 54, no. 3: 283–291. 10.1007/s10329-013-0347-5. 23455845

[ajp70069-bib-0033] León, J. , C. Thiriau , C. Crockford , and K. Zuberbühler . 2023. “Comprehension of Own and Other Species' Alarm Calls in Sooty Mangabey Vocal Development.” Behavioral Ecology and Sociobiology 77, no. 5: 56. 10.1007/s00265-023-03318-6. 37234238 PMC10205891

[ajp70069-bib-0034] Liebal, K. , B. Waller , A. Burrows , and K. E. Slocombe . 2013. Primate Communication: A Multimodal Approach. Cambridge. Cambridge University Press. 10.1177/147470491301100305 PMC10480985 23864293

[ajp70069-bib-0035] Ligges, U. , S. Krey , O. Mersmann , and S. Schnackenberg (2023). tuneR: Analysis of Music and Speech. https://CRAN.R-project.org/package=tuneR.

[ajp70069-bib-0036] Mann, H. B. , and D. R. Whitney . 1947. “On a Test of Whether One of Two Random Variables Is Stochastically Larger Than the Other.” Annals of Mathematical Statistics 18, no. 1: 50–60.

[ajp70069-bib-0037] Mitani, J. C. , and T. Nishida . 1993. “Contexts and Social Correlates of Long‐Distance Calling by Male Chimpanzees.” Animal 45: 735–746.

[ajp70069-bib-0038] Nowicki, S. , and W. A. Searcy . 2014. “The Evolution of Vocal Learning.” Current Opinion in Neurobiology 28: 48–53. 10.1016/j.conb.2014.06.007. 25033109

[ajp70069-bib-0039] Perry, S. 2012. The Behavior of Wild White‐Faced Capuchins. Demography, Life History, Social Relationships, and Communication. Advances in the Study of Behavior (44, 1st ed. Elsevier Inc. 10.1016/B978-0-12-394288-3.00004-6.

[ajp70069-bib-0040] Perry, S. , M. Baker , L. Fedigan , et al. 2003. “Social Conventions in Wild White‐Faced Capuchin Monkeys: Evidence for Traditions in a Neotropical Primate.” Current Anthropology 44, no. 2: 241–268. 10.1086/345825.

[ajp70069-bib-0041] Perry, S. E. , B. J. Barrett , and I. Godoy . 2017. “Older, Sociable Capuchins (*Cebus capucinus*) Invent More Social Behaviors, but Younger Monkeys Innovate More in Other Contexts.” Proceedings of the National Academy of Sciences 114, no. 30: 7806–7813. 10.1073/pnas.1620739114. PMC5544268 28739946

[ajp70069-bib-0042] Pistorio, A. L. , B. Vintch , and X. Wang . 2006. “Acoustic Analysis of Vocal Development in a New World Primate, the Common Marmoset (*Callithrix Jacchus*).” Journal of the Acoustical Society of America 120, no. 3: 1655–1670. 10.1121/1.2225899. 17004487

[ajp70069-bib-0043] R Core Team . 2023. R: A Language and Environment for Statistical Computing. R Foundation for Statistical Computing. URL: https://www.R-project.org/.

[ajp70069-bib-0044] Seyfarth, R. M. , and D. L. Cheney . 1986. “Vocal Development in Vervet Monkeys.” Animal Behaviour 34, no. 6: 1640–1658. 10.1016/S0003-3472(86)80252-4.

[ajp70069-bib-0045] Snowdon, C. T. 2018. “Cognitive Components of Vocal Communication: A Case Study.” Animals: An Open Access Journal from MDPI 8, no. 7: 126. 10.3390/ani8070126. 30041425 PMC6070781

[ajp70069-bib-0046] Steinberg, D. L. , J. W. Lynch , and E. A. Cartmill . 2022. “A Robust Tool Kit: First Report of Tool Use in Captive Crested Capuchin Monkeys (*Sapajus robustus*).” American Journal of Primatology 84, no. 11: 1–15. 10.1002/ajp.23428. 35942577

[ajp70069-bib-0047] Sugiura, H. 2001. “Vocal Exchange of Coo Calls in Japanese Macaques.” In Primate Origins of Human Cognition and Behavior, edited by T. Matsuzawa , 135–154. Springer.

[ajp70069-bib-0048] Taylor, A. M. , and D. Reby . 2010. “The Contribution of Source‐Filter Theory to Mammal Vocal Communication Research.” Journal of Zoology 280, no. 3: 221–236. 10.1111/j.1469-7998.2009.00661.x.

[ajp70069-bib-0049] Tinsley Johnson, E. , M. E. Benítez , A. Fuentes , et al. 2020. “High Density of White‐Faced Capuchins (*Cebus capucinus*) and Habitat Quality in the Taboga Forest of Costa Rica.” American Journal of Primatology 82, no. 2: 1–12. 10.1002/ajp.23096. 31976575

[ajp70069-bib-0050] Tomasello, M. 2008. “Primate Intentional Communication.” In Origins of Human Communication, 13–56. MIT Press.

[ajp70069-bib-0051] Wilbrecht, L. , and F. Nottebohm . 2003. “Vocal Learning in Birds and Humans.” Mental Retardation and Developmental Disabilities Research Reviews 9, no. 3: 135–148. 10.1002/mrdd.10073. 12953292

[ajp70069-bib-0052] Zimbler‐DeLorenzo, H. S. , and A. I. Stone . 2011. “Integration of Field and Captive Studies for Understanding the Behavioral Ecology of the Squirrel Monkey (*Saimiri sp*.).” American Journal of Primatology 73, no. 7: 607–622. 10.1002/ajp.20946. 21404315

[ajp70069-bib-0053] Zimmermann, E. , and C. Lerch . 1993. “The Complex Acoustic Design of an Advertisement Call in Male Mouse Lemurs (*Microcebus murinus*, Prosimii, Primates) and Sources of Its Variation.” Ethology 93, no. 3: 211–224. 10.1111/j.1439-0310.1993.tb00990.x.

[ajp70069-bib-0055] Zuberbühler, K. 2017. “The Primate Roots of Human Language.” In Primate Hearing and Communication, edited by R. M. Quam , J. M. Ramsier , R. R. Fay , and A. N. Popper , 175–200. Springer. 10.1007/978-3-319-59478-1_7.

[ajp70069-bib-0054] Zuberbühler, K. , K. Ouattara , A. Bitty , A. Lemasson , and R. Noë . 2009. “The Primate Roots of Human Language: Primate Vocal Behaviour and Cognition in the Wild.” In Becoming Eloquent: Advances in the Emergence of Language, Human Cognition, and Modern Cultures, edited by F. d'Errico and J.‐M. Hombert , 235–266. John Benjamins. 10.1075/z.152.10ch9.

