# Supplementary material for: The Ontogeny of “Twitter” Calls in White‐Faced Capuchins (Cebus imitator): Usage, Context, and Acoustic Structure
Source: Am J Primatol. 2025 Aug 26;87(8):e70069. doi: 10.1002/ajp.70069 (PMC12379072; doi:10.1002/ajp.70069)
Supplement: Supplementary file 1 — Supplementary_Information [file AJP-87-e70069-s001.docx]

Supplementary Information

The ontogeny of ‘twitter’ calls in white-faced capuchins (*Cebus imitator*): usage, context, acoustic structure.

**Authors**: Nicole Guisneuf, Juan Carlos Ordoñez, Marcela E. Benítez, Jacinta, C. Beehner, Thore J. Bergman

Nicole Guisneuf

[nickigui@umich.edu](mailto:nickigui@umich.edu)

University of Michigan
East Hall, 530 Church St
Ann Arbor, MI 48109

To examine whether age predicted the context of twitters, we created four negative binomial models, one for each behavioral state: social, foraging, travelling, resting. For each model, we used the total twitter count in that state as the outcome, with age, and time spent in that state as the predictors. (formula: Count of twitters in a social state ~ age group + time spent in a social state). This was to test whether age group predicted the total number of twitters, while accounting for the time spent in that behavioral state.

Infants and juveniles both produced significantly more twitters in a social state, compared to adults, while accounting for time spent in a social state (infants: p < .001; juveniles: p = 0.020, full results in Table S1). This model was the best fit in an AIC comparison, with the lowest AIC value and 93% of the weight. Infants and juveniles both produced significantly more twitters in a foraging state, compared to adults, while accounting for time spent foraging (infants: p = 0.002; juveniles: p = 0.034). This model was also the best fit in an AIC comparison with the lowest AIC value and 96% of the weight. For travel twitters, infants did not have significantly different twitter counts to adults (p = 0.551), but juveniles did twitter more than adults, while accounting for time spent in a travel state (p = 0.551). However, the AIC comparison showed that this model was not substantially better than model 1 which included age but not time spent travelling (weights: model 2: 75%, model 1: 25%), making the results less reliable, since the fact that adults spend more time traveling may be influencing results. Finally, for resting twitters, infants did not have significantly different twitter counts to adults (p = 0.551), but juveniles did (p = 0.017). The AIC comparison showed this model had 78% of the weight and model 1 without time spent resting had 22%, so again the positive association is unreliable. Taken together, these model results indicate that immature monkeys use twitters socially, and during foraging, more than adults. However, because infants and juveniles had higher twitter rates overall, these results don’t necessarily make sense.

**TABLE S1**: Results of glmmTMB on the effects of age group and time spent in each state, on twitter count in specific behavioral state.

| **Foraging state** | |  | |  |  |  |  |  |
| --- | --- | --- | --- | --- | --- | --- | --- | --- |
|  | Effect | | Estimate | SE | 95% CI |  | Z value | P |
|  | intercept | | -0.99 | 0.5 | -2.06 | -0.02 | -1.99 | 0.05* |
|  | age - infant | | 1.89 | 0.61 | 0.74 | 3.16 | 3.12 | 0.002** |
|  | age - juvenile | | 0.9 | 0.43 | 0.08 | 1.78 | 2.12 | 0.03* |
|  | Time in foraging state | | 0.01 | 0.001 | 0.008 | 0.01 | 8.17 | <0.001*** |
| **Social state** | | |  |  |  |  |  |  |
|  | Effect | | Estimate | SE | 95% CI |  | Z value | P |
|  | intercept | | -1.04 | 0.48 | -1.1 | -0.12 | -2.17 | 0.03* |
|  | age - infant | | 2.01 | 0.59 | 0.92 | 3.23 | 3.41 | 0.0006*** |
|  | age - juvenile | | 1.04 | 0.45 | 0.17 | 1.92 | 2.32 | 0.02* |
|  | Time in social state | | 0.02 | 0.002 | 0.01 | 0.02 | 6.78 | <0.001*** |
| **Resting state** | | |  |  |  |  |  |  |
|  | Effect | | Estimate | SE | 95% CI |  | Z value | P |
|  | intercept | | -1.39 | 0.86 | -3.18 | 0.32 | -1.61 | 0.11 |
|  | age - infant | | -0.29 | 0.48 | -3.66 | 2.5 | -0.19 | 0.85 |
|  | age - juvenile | | 1.86 | 0.86 | 0.36 | 3.48 | 2.17 | 0.03* |
|  | Time in resting state | | 0.02 | 0.006 | 0.003 | 0.03 | 2.73 | 0.006** |
| **Travel state** | | |  |  |  |  |  |  |
|  | Effect | | Estimate | SE | 95% CI |  | Z value | P |
|  | intercept | | -1.89 | 1.01 | -4.3 | 0.19 | -1.87 | 0.06 . |
|  | age - infant | | 0.8 | 1.35 | -2.01 | 3.76 | 0.6 | 0.55 |
|  | age - juvenile | | 2.18 | 0.91 | 0.56 | 4.04 | 2.38 | 0.02* |
|  | Time in travel state | | 0.04 | 0.01 | 0.01 | 0.07 | 2.85 | 0.004** |

**Table S2**: Results of Mann-Whitney U Test comparing infants/juveniles with adults, dividing actor and recipient. Actor = behavior was done by focal individual; recipient = another individual did the behavior towards focal individual.

|  |  |  |  |  |
| --- | --- | --- | --- | --- |
|  |  | **W value** | **P value** |  |
| Approach | *actor* | 42.5 | 0.03 | * |
|  | *recipient* | 47 | 0.055 | . |
| Leave | *actor* | 55 | 0.133 |  |
|  | *recipient* | 70 | 0.44 |  |
| Groom | *actor* | 112 | 0.144 |  |
|  | *recipient* | 69 | 0.335 |  |
| Aggress | *actor* | 94 | 0.558 |  |
|  | *recipient* | 60 | 0.143 |  |
